# DDT (Dichlorodiphenyltrichloroethane): MAK Value Documentation, addendum – Translation of the German version from 2023

**DOI:** 10.34865/mb5029e10_2ad

**Published:** 2025-06-30

**Authors:** Andrea Hartwig, MAK Commission

**Affiliations:** 1 Institute of Applied Biosciences. Department of Food Chemistry and Toxicology. Karlsruhe Institute of Technology (KIT) Adenauerring 20a, Building 50.41 76131 Karlsruhe Germany; 2 Permanent Senate Commission for the Investigation of Health Hazards of Chemical Compounds in the Work Area. Deutsche Forschungsgemeinschaft, Kennedyallee 40, 53175 Bonn, Germany. Further information: Permanent Senate Commission for the Investigation of Health Hazards of Chemical Compounds in the Work Area | DFG

**Keywords:** DDT, Pestizid, Insektizid, Toxizität, Bewertung, DDT, pesticide, insecticide, toxicity, evaluation

## Abstract

DDT [50-29-3] and DDT preparations are no longer approved in the European Union or in Germany. The previous documentation does not reflect the current data situation of the substance. The MAK Commission decided that a new evaluation is not of high priority. The MAK value and the other classifications are therefore suspended and the substance is listed in the Section II c of the List of MAK and BAT Values for substances no longer evaluated.

**Table:** 

**MAK value**	**see Section II c of the List of MAK and BAT Values**
**Peak limitation**	**–**
	
**Absorption through the skin**	**–**
**Sensitization**	**–**
**Carcinogenicity**	**–**
**Prenatal toxicity**	**–**
**Germ cell mutagenicity**	**–**
	
**BAT value**	**–**
	
Synonyms	clofenotane 4,4ʹ-dichlorodiphenyltrichloroethane
Chemical name (IUPAC)	1,1,1-trichloro-2,2-bis(4-chlorophenyl)ethane
CAS number	50-29-3
Molecular formula	C_14_H_9_Cl_5_
Molar mass	354.49 g/mol
Melting point	108.5 °C (IFA 2022)
Vapour pressure at 20 °C	2.13 × 10^–7 ^hPa (NCBI 2023)
log K_OW_ at 20 °C	6.36 (IFA 2022)
Solubility	0.006 mg/l water (IFA 2022)

A MAK value of 1 mg/m^3^ was established for the inhalable fraction of DDT in 1969 and the substance was classified in Peak Limitation Category II with an excursion factor of 8 in 2002. In 1966, DDT was designated with an “H” (for substances which can be absorbed through the skin in toxicologically relevant amounts) due to the hazard posed by percutaneous absorption. To date, the only MAK documentation for DDT concerned peak limitation (Greim 2002, available in German only).

From 1946 to 1972, DDT was the most widely applied insecticide in agriculture and forestry and was used particularly for combating malaria (Umweltprobenbank des Bundes 2022). The substance acts as an endocrine disruptor (AERU 2022). DDT is one of the original 12 persistent organic pollutants (POP) that were banned from worldwide production, sale and use under the Stockholm Convention (or “POP Convention”) of 22 May 2001 that came into force on 17 May 2004 (UBA 2021). In the European Union, the use of DDT is prohibited under Regulation (EC) No 1107/2009 concerning the placing of plant protection products on the market (European Commission 2022 b; European Parliament and European Council 2009, 2019) and the substance has been placed under an export ban (European Commission 2022 a). In the Federal Republic of Germany, the use and production of DDT and formulations containing DDT have been prohibited since 1972 (BRD 1972). In the German Democratic Republic, DDT continued to be used in large quantities in the 1980s to control the bark beetle (Umweltprobenbank des Bundes 2022) until the substance was banned in 1988 (BVL 2010).

A re-evaluation of DDT is not a priority. Therefore, the MAK value, the peak limitation and the “H” designation have been withdrawn and the substance has been allocated to Section II c of the List of MAK and BAT Values (DFG 2022). This section lists substances for which the previous MAK values, designations and classifications have been withdrawn and which are no longer being reviewed at present.
